# Arginine Alters miRNA Expression Involved in Development and Proliferation of Rat Mammary Tissue

**DOI:** 10.3390/ani11020535

**Published:** 2021-02-19

**Authors:** Gang Zhou, Qiaoyun Xu, Feifan Wu, Mengzhi Wang, Lianmin Chen, Liangyu Hu, Jingwen Zhao, Juan J. Loor, Jun Zhang

**Affiliations:** 1 Huaiyin Institute of Agricultural Sciences in Xuhuai Regio, Huaian 223000, China; yzdxzg@163.com; 2 College of Animal Science and Technology, Yangzhou University, 88 South University Ave., Yangzhou 225009, China; jingkenxu@163.com (Q.X.); wffyzu@126.com (F.W.); mzwang@yzu.edu.cn (M.W.); lianminchen@yeah.net (L.C.); huyu1032@163.com (L.H.); zhaojingwen79@163.com (J.Z.); 3 Department of Genetics, University of Groningen, University Medical Center Groningen, 9713 AV Groningen, The Netherlands; 4 Department of Pediatrics, University of Groningen, University Medical Center Groningen, 9713 AV Groningen, The Netherlands; 5 Department of Animal Sciences and Division of Nutritional Sciences, University of Illinois, 1207 W Gregory Drive, Urbana, IL 61801, USA; jloor@illinois.edu

**Keywords:** arginine, rat, mammary, miRNAs

## Abstract

**Simple Summary:**

MiRNAs are small noncoding RNAs that regulate a variety of developmental and physiological processes, with many having well-defined developmental and cell-type specific expression patterns. Aspects of the cell cycle such as cell differentiation, proliferation and apoptosis can be regulated by miRNA, underscoring an unexplored link between arginine supply and mammary tissue function during lactation. The specific objective of the present study was to determine miRNA profiles in mammary tissue at the end of lactation in response to enhanced dietary supply of arginine. Our results indicate that arginine may potentially be involved in the development of rat mammary glands through miRNA.

**Abstract:**

This study was designed to determine the effects of dietary arginine on development and proliferation in rat mammary tissue through changes in miRNA profiles. Twelve pregnant Wistar rats were allocated randomly to two groups. A basal diet containing arginine or the control diet containing glutamate on an equal nitrogen basis as the arginine supplemented diet were used. The experiment included a pre-experimental period of four days before parturition and an experimental period of 17 days after parturition. Mammary tissue was collected for histology, RNA extraction and high-throughput sequencing analysis. The greater mammary acinar area indicated that arginine supplementation enhanced mammary tissue development (*p* < 0.01). MicroRNA profiling indicated that seven miRNA (miR-206-3p, miR-133a-5p, miR-133b-3p, miR-1-3p, miR-133a-3p, miR-1b and miR-486) were differentially expressed in response to Arginine when compared with the glutamate-based control group. In silico gene ontology enrichment and KEGG pathway analysis revealed between 240 and 535 putative target genes among the miRNA. Further verification by qPCR revealed concordance with the differential expression from the sequencing results: 17 of 28 target genes were differentially expressed (15 were highly expressed in arginine and 2 in control) and 11 target genes did not have significant difference in expression. In conclusion, our study suggests that arginine may potentially regulate the development of rat mammary glands through regulating miRNAs.

## 1. Introduction

It is well known that the mammary gland is a central organ for milk synthesis, thus, development and metabolism of the mammary gland will affect the production of milk components such as fat and protein. The uptake and utilization of amino acids by the mammary gland also plays an important role in milk protein synthesis and milk yield [1,2]. Studies have shown that amino acids are important precursors and signal molecules for the synthesis of milk proteins. Among them, 90% of milk proteins are synthesized from amino acids in mammary epithelial cells [3]. Amino acids can not only regulate transcription and translation of milk proteins through a variety of signaling cascades [4], but also are closely related to the growth and development of the mammary gland and lactation performance [2]. The proliferation and differentiation of mammary are affected by many factors, such as hormones, vitamins, amino acids and trace elements [5]. These factors also affect the proliferation and differentiation of mammary epithelial cells. For example, arginine, as an essential amino acid for newborns and conditionally-essential for adult mammals, is considered a “functional” amino acid in the body. It does not only promote the proliferation and differentiation of mammary cells, but also promotes protein biosynthesis [6].

Compared with other organs, arginine is not only a substrate for milk protein synthesis, but also is involved in the formation of mammary glands in cells. As a functional amino acid, arginine can promote cell proliferation in two ways: as a semi-essential amino acid for cell growth; or through promoting the synthesis of nitric oxide [7,8]. Arginine can regulate mammary cell growth and lactation performance through nitric oxide synthesis [2]. A low concentration of nitric oxide can regulate cell proliferation-related gene expression and promote cell growth, while high concentrations of nitric oxide can inhibit cell proliferation [9]. In a recent experiment involving arginine supply and casein synthesis in mammary epithelial cells, Chen et al. [10] found that arginine could promote the proliferation of bovine mammary epithelial cells in vitro. The study shows that enhanced supply of arginine can activate mechanistic target of rapamycin (mTOR) and signal transducer and activator of transcription (STAT) signaling pathways to improve milk protein synthesis [11].

MicroRNAs (miRNAs) are small non-coding RNAs that regulate a variety of developmental and physiological processes. Many of them have specific developmental and cell type-specific expression patterns. It is estimated that 30% of miRNAs are involved in the regulation of endogenous gene expression, thus, they play an important role in various biological processes. Specifically, miRNAs directly regulate the expression of milk component coding genes, cell differentiation and proliferation [12,13]. To our knowledge, the role of arginine supply in mammary development and lactation has not been confirmed. Therefore, in order to understand better the relationship between arginine supply and lactation functions of the mammary gland, we hypothesized that arginine may regulate functional genes associated with lactation and mammary tissue development by regulating the expression of miRNAs. The aim of this study was to increase the supply of arginine and determine the distribution of miRNA in the mammary gland at the end of lactation. In addition, histological examination was performed to assess morphological changes associated with arginine supply and changes in miRNA profile.

## 2. Materials and Methods

### 2.1. Experimental Animals and Design

All animal procedures were approved by the Yangzhou University Animal Care and Use Committee of Jiangsu Province (China) (the code is SYXK (Su) 2017-0044). In this experiment, rats were killed by cervical dislocation according to Carbone L., et al. [14]. A littermate-control test was used and 12 healthy pregnant Wistar rats (the Animal Care and Use Committee of Yangzhou University, China) at the same physiological stage (weighted 260 ± 10 g) were allocated randomly in equal numbers to two groups, a control and arginine-supplemented group. Individual batch, routine feeding as well as management and immunization programs were conducted. Natural lighting, ventilation, free feeding and drinking were provided.

The basal diet fed was AIN-93G [15] (Nantong Trophy Co., Ltd. Nantong, China). The basic dietary formula and nutrient content of the experiment group (basal diet plus 1-fold content of arginine) and the control group (as nitrogen balance is an important issue in nutritional study, to avoid the potential bias of unbalanced nitrogen amount, we added the non-essential and most common amino acid glutamate into basal diets with the same content of nitrogen as arginine) are reported in Table 1. The experimental period included a pre-experimental period (four days before parturition) and the experimental period of 17 days after parturition.

#### Verification of miRNA and Target Genes Expression by qPCR

RNA samples from 12 rats for miRNA-seq were further used to validate differential miRNAs identified by miRNA-seq. Unlike the polled RNA samples used in miRNA-seq, RT-qPCR was performed for 12 rats separately. The miRNAs expression was validated by poly (A)-tailed qPCR. Firstly, reverse transcription was carried out from 0.5 μg of the total RNA samples using miScript II RT Kit (Cat. # 218160, Qiagen, Hilden, Germany) with miScript HiFlex buffer, and then the synthesized cDNA was used as a template for qPCR reactions. qPCR reactions were performed using a 7500 Real-Time PCR System (Applied Biosystems, Foster City, CA, USA) using an miScript SYBR Green PCR Kit (Cat. # 218073, Qiagen, Hilden, Germany) according to the manufacturer’s instructions. This system was reported as showing relatively high accuracy and high sensitivity among several quantification methods [16]. The following real-time PCR protocol was used: initial activation of HotStarTaq DNA Polymerase (from miScript kit; 95 °C, 15 min); 40 cycles of denaturation (94 °C, 15 s), annealing (55 °C, 30 s) and extension (70 °C, 34 s); and melting curve analysis. The primers used for the target miRNA are designed by Shanghai Biotechnology Corporation (China) and listed in Table S1.

For qPCR experiments of mRNAs, reverse-transcription was performed using a FastQuant RT Kit (with gDNase) (Cat. # KR106, Tiangen, Beijing, China). qPCR was performed using SYBR SuperReal Premix plus (Cat. # FP205, Tiangen, Beijing, China) on a 7500 Real-Time PCR System (Applied Biosystems, Foster City, CA, USA) according to the manufacturer’s protocol. The following real-time PCR protocol was used: polymerase activation (95 °C, 15 min), 40 cycles of denaturation (95 °C, 10 s), annealing and extension (60 °C, 32 s) and melting curve analysis. The primers used for the target mRNA are designed by Intivrogen (Shanghai, China) and listed in Table S2.

The data were analyzed using 7500 Software ver. 2.0.4. (Applied Biosystems, Foster City, CA, USA) with the fixed Ct setting (ΔRn = 0.2) to assign baseline values and the threshold for Ct determination. The relative expression of miRNA and mRNA was normalized to U6 and GAPDH expression and relative to the control. Expression data was calculated by using the 2^−ΔΔCt^ method as described previously [17]. Statistical analysis was carried out via *T*-Test using SPSS16.0. A *p*-value ≤ 0.05 was considered significant unless otherwise stated.

### 2.2. Collection of Rat Mammary Gland Tissue

At the end of the experimental feeding, the mammary gland tissues of the 12 rats were collected. A subsample of tissues was directly cryopreserved in liquid nitrogen and sent to Shanghai Bohao Biotechnology Co., Ltd. (Shanghai, China). for miRNA extraction and high-throughput sequencing in September 2014 while other tissues were rinsed with normal saline and use for mammary histological sections fixed in formaldehyde.

### 2.3. Mammary Histological Section Preparation and Acinar Area Measurement

The paraffin-fixed blocks were serially sectioned into 6–7 μm coronal slices and stored at −20 °C. For routine histological studies, paraffin sections were stained with hematoxylin and eosin [18]. Hematoxylin–eosin stained sections were analyzed by light microscopy using an Olympus fluorescence microscope (Olympus, Tokyo, Japan). For each rat, we randomly made three HE-staining images. Mammary acinar areas were measured using digital imaging system software MC30 (Mingmei, Guangzhou, China) and all acinar areas were recorded from each rat (1200 acinar areas in total). One-way ANOVA in SPSS 16.0 was conducted to determine differences from the control group. Significant difference was declared at *p* < 0.05.

### 2.4. Mammary RNA Extraction and Sequencing Library Construction

Quality inspection of total RNA extracted from rat mammary gland samples was conducted using the Qubit 2.0 fluorescent quantitative instrument and Agilent Bioanalyzer 2100 (Agilent Technologies Santa Clara, Santa Clara, CA, USA). Total RNA was purified according to the ‘TruSeq Small RNA Sample Preparation Guide’ and ligated at the 3′ and 5′ end, respectively. After reverse transcription, amplification and cDNA purification, the sequencing library was constructed. The library was tested for concentration using Qubit^®^ 2.0 Fluorometer and the Agilent 2100 was used to determine the size of the library.

### 2.5. Cluster Generation and High-Throughput Sequencing Analysis

Cluster generation and sequencing primer hybridization were completed on cBot of the Illumina GA IIx Sequencer according to the processes indicated in the cBot User Guide. After preparing sequencing reagents according to the Genome Analyzer IIx User Guide, the Illumina Single Read Flow Cell V4 mounted on a cluster was tested (GA IIx, Illumina). Single-read 1 × 50 nt multiplex was used to perform single-read. Sequencing processes and real-time data analysis were controlled by Illumina’s Data Collection Software.

### 2.6. Data Comparison and Classification

The pretreatment of sequencing primary reads and removing of the linker sequences and low-quality sequences (including fuzzy base N and bases less than 10 in mass and 18 nt in length) was conducted using fastx (fastx_toolkit-0.0.13.2). The pre-processed sequences were compared with the Sanger miRbase [19] database (21.0) (no mismatch allowed) and with ncRNA [20], piRNA [21] and Rfam [22] (two base mismatches to the target sequence and shorter or longer two bases at each end than the target sequence were allowed).

### 2.7. Saturation Analysis

The amount of unique smallRNAs, annotated smallRNAs, annotated reads and microRNAs identified in miRBase were examined as the amount of sequencing increased.

### 2.8. Differential Expression Analysis of miRNAs and Target Gene Prediction

After the miRNA reads were quality checked, clean reads were used to calculate the abundance of miRNAs which were recorded as TPM (Transcripts Per Kilobase Million). We first divided the read counts by the length of each gene in kilobases (RPK). Next, we counted up all the RPK values in a sample and divided this number by 1,000,000. The DEGseq R language package in conjunction with a perl script were used to compare sample expression differences and the logarithm of relative changes in log base 2 taken; after comparing with the *p*-value by fisher test, an FDR adjustment was performed.

The miRanda software was used to predict the differentially expressed miRNAs sequences based on 2.3.4 novel miRNAs to predict possible target gene sites of corresponding rat genomic cDNA sequences.

### 2.9. Novel miRNA Prediction

The miRCat tool in the sRNA Toolkit package was used to predict novel miRNAs. The signature microarray structure of microRNA precursors could be used to forecast new microRNAs. After comparing the unanalyzed reads in the annotations (the sample name was _unannotated.csv) with the rat genome, the sequence was initially determined as a candidate for new microRNA through the folding model when it was located on the stem-loop structure.

### 2.10. High Throughput Sequencing Analysis

#### 2.10.1. RNA Quality Detection

The total amount of extracted RNA sample was above 50.70 µg and the A260/A280 values of RNA were between 2.08 and 2.13. The RNA Integrity Number (RIN) values of samples were between 7.8 and 9.6 and the 28S/18S between 0.7 and 1.7 (Agilent 2100 Bioanalyzer, Agilent Technologies Inc., Santa Clara, CA, USA). This was in line with further high-throughput sequencing requirements.

#### 2.10.2. Library Quality Control

The Qubit^®^ 2.0 Fluorometer and Agilent 2100 results showed that the Con./concentration (ng/μL) was between 0.422 and 0.728 while the Peak Length./specific peak length (bp) was between 143 and 153. Thus, the library could be sequenced.

#### 2.10.3. Saturation Analysis 

Sequencing results indicated that the number of original reads (raw reads) of each sample after sequencing were more than 20M; the ratio of each base whose mass was greater than 20 (Q20) was more than 98.47%. Thus, data were adequate for analysis.

## 3. Results

### 3.1. Effect on the Development of Mammary Tissue

**Figure 1:**
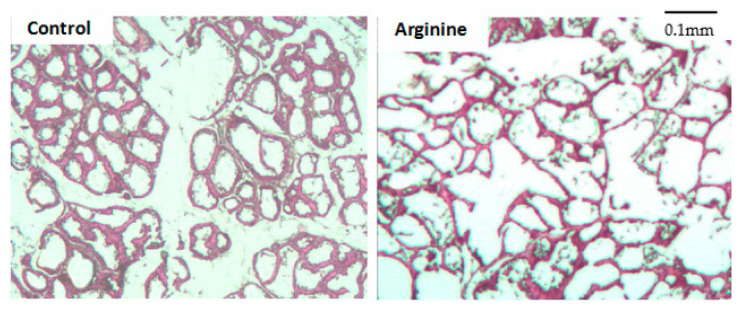
Rat mammary acinar histological section (400×).

**Figure 2:**
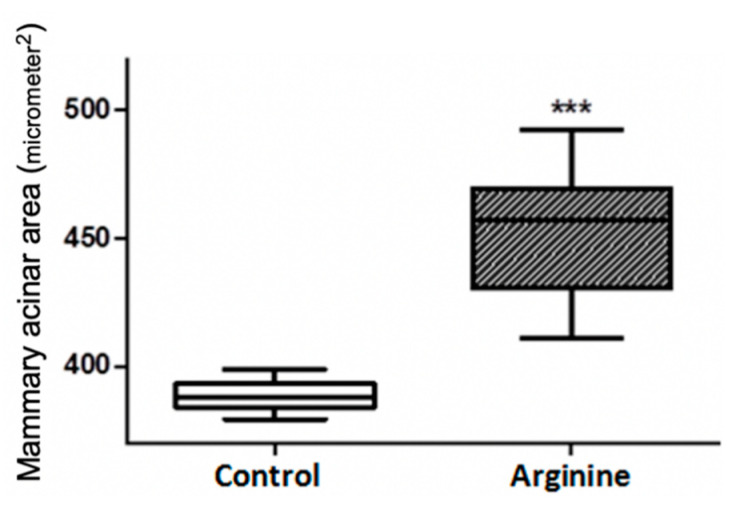
Comparison of mammary acinar area (um^2^) *** Represents *p* < 0.001.

Figure 1 depicts a mammary histological section of postnatal rats. The mammary acinus of the arginine group was more regular and consistent as well as relatively larger in sectional area while the control group was not uniform in size and relatively small in sectional area with partial incomplete development. By measuring and calculating the sectional acinar area of the two groups, we observed that sectional acinus area with dietary arginine was significantly larger than the control (*p* < 0.001) (Figure 2).

### 3.2. High Throughput Sequencing Analysis and RT-PCR Verification

**Figure 3:**
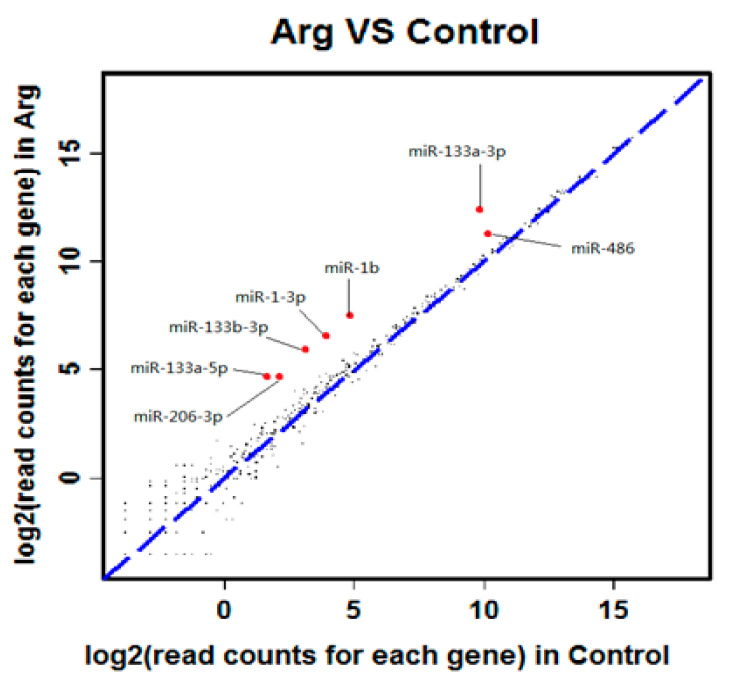
Differently expressed miRNAs (FC < 0.5 or FC > 2).

**Figure 4:**
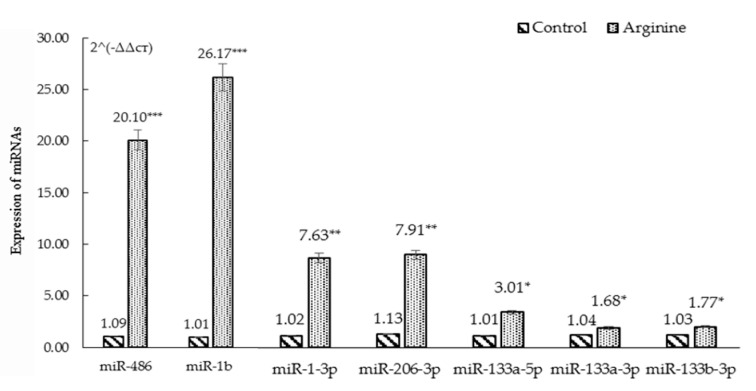
RT-PCR verification of differential expression of miRNAs screened by high throughput sequencing analysis (FC < 0.5 or FC > 2). * *p* < 0.05, ** *p* < 0.01, *** *p* < 0.001.

As shown in Table 2, 50 differentially expressed miRNAs (*p* < 0.001, FDR < 0.01) were obtained by primary screening at FDR < 0.01 (*p* < 0.001) in which 22 were down-regulated and 28 were up-regulated. Furthermore, seven miRNA (miR-206-3p, miR-133a-5p, miR-133b-3p, miR-1-3p, miR-133a-3p, miR-1b and miR-486) were screened by the secondary sequencing of FC > 2 or FC < 0.5 which were all up-regulated by arginine (Figure 3). Further verification by qPCR showed accordance with the differential expression of the sequencing (Figure 4).

### 3.3. Prediction and Enrichment Analysis of Target Genes of Differentially Expressed miRNA

**Figure 5:**
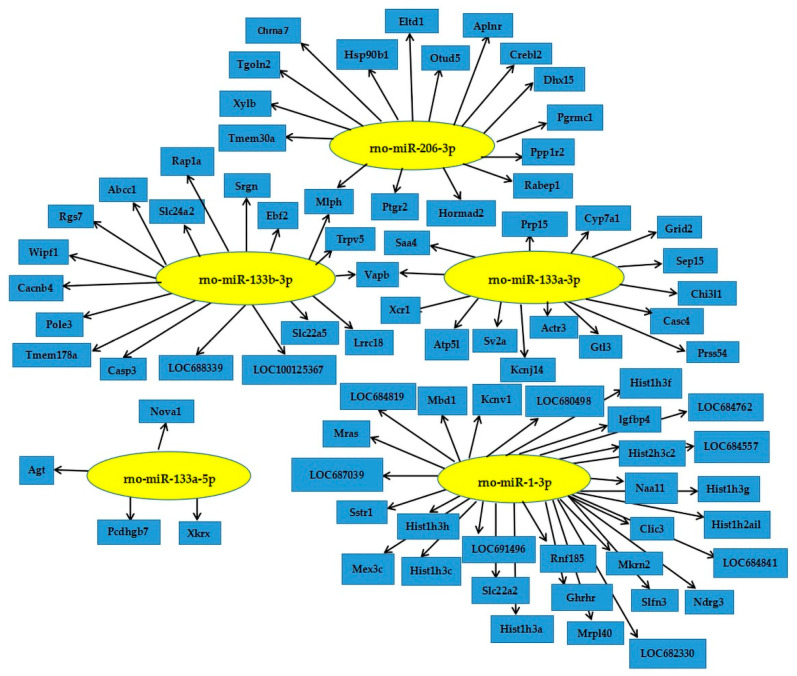
Regulatory pathway of differently expressed miRNAs.

The “SBC analysis wizard” was used to predict the target genes of differentially expressed miRNAs. Results indicated 44,142 and 55,401 in the control and arginine groups, respectively. The target genes of mir-133a-3p and mir-133b-3p were completely consistent via DAVID (https://david.ncifcrf.gov, version 6.7) and GO analysis (http://sas.ebioservice.com/portal/root/molnet_shbh/index.jsp, Intrated by SBC analysis system). In addition, the coincidence rate of mir-1-3p and mir-206-3p target genes were higher. Results of GO enrichment analysis were in line with KEGG pathway analysis. There were 240 out of 535 significantly correlative target genes through GO enrichment analysis and KEGG pathway analysis (Table 3). Figure 5 depicts a regulatory role of differentially expressed miRNAs in cell signaling and glucose metabolism and other pathways such as G-protein-coupled receptor signaling pathway, cell vesicle transport, Poly(A) RNA binding protein, RNA splicing, glucose pentose phosphate pathway, insulin secretion, cell adhesion and autophagy.

### 3.4. Target Gene Prediction of Differentially Expressed miRNAs and RT-PCR Validation

Seven differentially expressed miRNAs were used to predict their target genes via TargetScan, MiRanda, PicTar, MirTarget2 and PITA supported (http://prediction.ebioservice.com:8080/prediction/analysis) in which miR-486 and miR-1b failed to predict and screen for target genes. The 34 predicted target genes were detected by RT-PCR (Table 4). Differential miRNA targeted genes’ expression are shown in Table 5. In general, we observed that 17 out of 34 genes showed significant expression levels between the control and arginine groups. Notably, some of the differential expressed genes were known to be cell cycle related, indicating the potential roles of miRNAs in regulating mammary gland development.

## 4. Discussion

Rodent mammary tissue development begins at puberty, while the catheter system expands rapidly and forms immature acini [23]. The mammary gland grows rapidly in pregnancy, especially late pregnancy, and is accompanied with increased functional acini as well as volume of lobules. After a period of lactation, the size of the acini begins to shrink leading to mammary gland involution prior to a decrease in milk synthesis and cessation of lactogenesis. Pregnancy is a key period determining the amount of secretory cells and milk yield during lactation, and the performance of mature acini determines lactation capacity. In this study, we observed larger and more regular acini in the arginine group compared with the control group suggesting that this amino acid contributed to lactation by means of either stimulating acinus growth or maintenance of acinus morphology.

Available evidence demonstrates that the level of amino acids especially arginine uptake by mammary is much higher than the level secreted in milk [9,24]. Further meta-analysis by Lapierre proved that the average uptake: output ratio averages 2.45 no matter the protein sources or levels [25], which might indicate that the arginine that had been taken basically does not participate in the formation of structural protein of the mammary gland. Similarly, Mezl and Knox (1977) [26] observed a significant conversion of arginine to proline and glutamate in lactating rat mammary gland, with conversion increasing as milk production rose suggesting a dominant disposition of excess arginine in the gland. O’Quinn et al. (2002) [27] pointed out that proline, ornithine and urea were the major products of arginine catabolism in lactating porcine mammary tissue. Together, data suggested that most arginine from plasma to mammary cannot participate in milk synthesis or tissue accretion directly. However, the present study showed positively larger alveolar cells in mammary tissue in response to dietary arginine we hypothesized. Thus, this effect probably was not only caused directly by increased concentration of arginine but also indirectly through its metabolism. Arginine is the precursor of NO and polyamines which are bioactive molecules stimulating cell proliferation and migration, cell recombination, angiogenesis, dilatation and increased blood flow in mammary thereby increasing the uptake of nutrients [28]. Mateo et al. (2008) [29] found that supplementing L-arginine to the diets of primiparous sows could enhance milk production. Arginine-enriched or citrulline-abundant foods are supposed to have great potential to enhance NO production and cell proliferation in mammary tissue [7].

The miRNAs are a class of non-coding small RNA involved in post-transcriptional gene silencing to regulate transcription primarily via degradation of the complementary mRNA target and/or translational suppression. The development of mammary tissue is accompanied by morphological and transcriptional changes through the stages of lactation associated with differentially expressed miRNA. Thus, miRNA sequencing offers the opportunity to reveal the gene expression of an individual in a particular developmental period and in a given tissue. In other words, a miRNA will be assumed to play a regulatory role in a particular tissue or cell, or at the specific developmental stage if differential expression of the miRNA is associated with that condition. In this study, we observed seven miRNAs up-regulated via differential expression analysis and verified by qPCR including miR-206-3p, miR-133a-5p, miR-133b-3p, miR-1-3p, miR-133a-3p, miR-1b and miR-486, suggesting these miRNAs probably play roles in the well-developed acini cells when rats were fed arginine.

MicroRNAs are widely expressed at various stages of development of cells, tissue and organs, with their expression characterized by profiles called spatio-temporal or time-dependent patterns associated with specific developmental stages [30]. Such patterns indicate that they are required for tissue morphogenesis and cell differentiation in development and apoptosis. The expression pattern of miRNAs obtained throughout the murine mammary developmental stages including juvenile, puberty, mature virgin, pregnancy and lactation indicated that miRNAs are functionally involved in mammary gland development [31,32]. Although our observation of differential expression of miRNAs in this study did not relate to spatio-temporal expression patterns, the upregulation of the miR-133 family is consistent with the report of Wang and Li [27] that miR-133 and miR-133a-133b are up-regulated during pregnancy and lactation, which corresponds to the point of sampling in the current experiment.

Single tissue-specific miRNAs have been identified in specific mammalian organs such as heart, liver or brain [33]. Interestingly, all of the miRNAs up-regulated we observed in this study have been reported as highly-expressed in muscle and belong to mammalian skeletal and cardiac muscle miRNA, involved in proliferation and differentiation during muscle formation. MiR-1, miR-133 and miR-206, for instance, are collectively known as the “myomiRs” for their prevalence in skeletal and cardiac muscle [34,35,36]. MiR-1 and miR-133, which are clustered on the same chromosomal loci, are transcribed together in a tissue-specific manner during development [37]. Although miR-1 and -206 are closely related in terms of expression and function, they differ based on chromosomal loci, specific targets and individual transcriptional activation [36]. The function of these three miRNA families on muscle development could be generalized as both miR-1 and miR-206 promoting myoblast-to-myotube differentiation. In contrast, miR-133 promotes proliferation of myoblasts, and inhibits their differentiation.

There already have been studies evaluating miRNAs expression in the mammary gland [38,39], thus, our observations expand the recognition about these tissue-specific miRNAs which may have simplification for non-muscular tissue. In addition, miR-486, another tissue-specific miRNA up-regulated in this study, plays not only powerful roles in muscle cells, but also in regulation of angiogenic activity. MiR-486 was first found in human fetal liver [40], and it has been reported that it is an erythroid-related microRNA and may play important roles in regulation of hematopoiesis [41,42]. Thus, it could be possible that hematopoiesis regulated by miR-486 is correlated to function of NO generated from arginine.

Despite the few reports or evidence showing that the differentially expressed miRNAs mentioned above have a role in mammary tissue, the results of the GO and KEGG analyses in the current study indicated several significant enrichment pathways mainly regulated by the differentially expressed miRNAs including cell signal, glucose metabolism protein coupled receptor signaling pathway and cell vesicle transport, poly(A) RNA binding protein, RNA splicing, glucose pentose phosphate pathway and insulin secretion and cell adhesion, autophagy and other pathways.

In the present study, by means of predicting target genes of the five miRNAs via different web-based tools, we further observed 15 target genes upregulated significantly including Mkrn2, Slfn3, Mex3c, Rnf185, Ndrg3, Tgoln2, Dhx15, Slc22a5, Casp3, Tmem178a, E1td1 (Adgrl4), Hsp90b1, Tmem30a, Ppp1r2 and Agt and two genes, Atp51 and 15-Seq, downregulated by arginine supplementation. This further indicated the direction in which arginine affects specific genes based on miRNAs. These target genes represent various functions regulating the biological processes involved in growth, energy, immunity, nervous system and tumor development. Among these genes, some particular upregulated genes whose expression is associated with hematopoiesis and angiogenesis, for example, Eltd1 (Adgrl4) which was highly expressed in microvascular endothelium has been reported to contribute to the development and homeostasis of the vascular system [43]. Angiotensinogen (Agt) is a precursor of angiotensin II (Ang II), an octapeptide pressor hormone that has a key role in mediating vascular constriction and regulating salt and fluid homeostasis [44]. Other target genes else also have similar biological function including Makrin2 [45] and Ndrg3 [46]. Based on the upregulation of target genes, we propose that the physiological phenomenon of hematopoiesis and angiogenesis is a significant part of mammary tissue activated by metabolism of arginine. However, we also acknowledge that the prediction of miRNA target genes is based on several classical tools. Recently, new prediction tools like AmiRNA designer [47] based on the thermodynamic analysis of the native miRNA/miRNA* and miRNA/target duplexes that allow researchers to perform analysis of natural miRNAs is worthy to try in the future study.

## 5. Conclusions

In summary, the supplementation of arginine altered the expression level of seven miRNAs including miR-206-3p, miR-133a-5p, miR-133b-3p, miR-1-3p, miR-133a-3p, miR-1b and miR-486 in mammary tissue potentially via some unknown pathways. The miRNAs and target genes screened out in this study have an unclear relationship with the related physiology of mammary and lactation. Thus, further research is needed to focus on the mechanisms whereby miRNAs selected in this work affect mammary gland development by manipulating cell cycle and cell division in response to supply of arginine.

## Figures and Tables

**Table 1:** Composition of the experimental diets (g/kg).

Ingredients	Control Group	Experimental Group
Cornstarch	397.47	378.29
Casein (>85% protein)	200	200
Dextrinized cornstarch (90%–94% tetrasaccharides)	110.38	125.6
Sucrose	100	100
Soybean oil (no additives)	70	70
Fiber	50	50
Mineral mix (AIN-93G-MX)	35	35
Vitamin mix (AIN-93-VX)	10	10
L-Cystine	3	3
Choline bitartrate (41.1% choline)	2.5	2.5
Tert-butylhydroquinone	0.014	0.014
L-Arginine	0	6.4 *
Glutamic acid	21.62 **	0
Nutrient content		
Digestible energy (MJ/kg)	15.925	15.925
CP (%)	18.287	18.287
E/P	0.871	0.871
L-Arginine (%)	0.64	1.28
Glutamic acid (%)	5.55	3.63

*: 1-fold content of arginine in basal diets; **: The nitrogen addition of arginine in the glutamic acid-balanced test group.

**Table 2:** Differentially expressed miRNAs with different treatment by RNA-Seq screen.

No.	miRNA	Control Tpm	Arg Tpm	FC (Arg/Control)	*p*-Value	FDR	Expression Level
1	miR-181a-1-3p	613.34	422.53	0.69	2.2 × 10^−9^	4.1 × 10^−8^	down
2	miR-141-3p	21,066.09	14,791.93	0.70	0	0	down
3	miR-192-5p	903.41	654.03	0.72	1.9 × 10^−10^	3.8 × 10^−9^	down
4	miR-200a-3p	2234.33	1643.09	0.74	8.3 × 10^−22^	3.0 × 10^−20^	down
5	miR-181c-5p	2070.42	1531.36	0.74	1.2 × 10^−19^	3.7 × 10^−18^	down
6	miR-200b-3p	4278.14	3427.11	0.80	7.8 × 10^−23^	3.3 × 10^−21^	down
7	miR-429	2578.68	2074.59	0.80	6.8 × 10^−14^	1.7 × 10^−12^	down
8	miR-101a-3p	5917.868	5023.77	0.85	3.0 × 10^−18^	8.6 × 10^−17^	down
9	let-7f-5p	17,008.48	14,530.47	0.85	1.9 × 10^−46^	1.1 × 10^−44^	down
10	let-7g-5p	3540.63	3050.85	0.86	7.9 × 10^−10^	1.5 × 10^−8^	down
11	miR-148a-5p	7142.35	6155.98	0.86	2.3 × 10^−18^	7.0 × 10^−17^	down
12	miR-181a-5p	48,487.41	42,283.78	0.87	0	0	down
13	miR-186-5p	4608.16	4080.20	0.89	6.8× 10^−9^	1.1 × 10^−7^	down
14	miR-26b-5p	2903.73	2592.21	0.89	1.7 × 10^−5^	2.7 × 10^−4^	down
15	miR-148a-3p	237,987.63	214,042.02	0.90	0	0	down
16	miR-31a-5p	2602.24	2368.22	0.91	6.5 × 10^−4^	7.4 × 10^−3^	down
17	miR-30e-5p	5237.25	4815.07	0.92	1.4 × 10^−5^	2.2 × 10^−4^	down
18	let-7i-5p	3746.97	3451.53	0.92	3.3 × 10^−4^	4.0 × 10^−3^	down
19	let-7a-5p	4848.41	4531.16	0.93	6.6 × 10^−4^	7.3 × 10^−3^	down
20	miR-30a-5p	28,750.68	27,008.46	0.94	6.2 × 10^−15^	1.6 × 10^−13^	down
21	miR-375-3p	43,642.12	41,710.08	0.96	8.5 × 10^−13^	2.0 × 10^−11^	down
22	miR-26a-5p	54,560.24	53,471.05	0.98	1.2 × 10^−4^	1.5 × 10^−4^	down
23	miR-10a-5p	36,589.32	37,617.76	1.03	5.0 × 10^−4^	5.9 × 10^−3^	up
24	miR-30d-5p	9351.35	9923.60	1.06	7.7 × 10^−5^	1.0 × 10^−3^	up
25	miR-27b-3p	13,604.16	14,733.03	1.08	6.6 × 10^−11^	1.4 × 10^−9^	up
26	let-7c-5p	4151.56	4503.59	1.08	2.5 × 10^−4^	3.1 × 10^−4^	up
27	miR-151-3p	3099.21	3377.26	1.09	8.1 × 10^−4^	8.4 × 10^−4^	up
28	miR-126a-5p	6980.50	7703.56	1.10	5.7 × 10^−9^	1.0 × 10^−7^	up
29	miR-146b-5p	8007.73	8901.84	1.11	1.7 × 10^−11^	3.8 × 10^−10^	up
30	miR-99b-5p	1625.39	1824.40	1.12	9.5 × 10^−4^	9.6 × 10^−3^	up
31	miR-205	1579.97	1785.98	1.13	5.2 × 10^−3^	6.0 × 10^−3^	up
32	miR-92a-3p	1888.21	2152.01	1.14	4.8 × 10^−5^	6.8 × 10^−4^	up
33	miR-191a-5p	8581.33	9838.75	1.15	7.2 × 10^−20^	2.4 × 10^−18^	up
34	miR-22-3p	33,500.82	38,702.25	1.16	1.1 × 10^−83^	8.2 × 10^−82^	up
35	miR-10b-5p	168,843.19	196,136.60	1.16	0	0	up
36	miR-181b-5p	792.72	935.12	1.18	7.5 × 10^−4^	8.0 × 10^−3^	up
37	miR-199a-3p	1008.57	1203.49	1.19	4.4 × 10^−5^	6.4 × 10^−4^	up
38	miR-126a-3p	1729.27	2068.87	1.20	5.7 × 10^−8^	9.4 × 10^−7^	up
39	miR-16-5p	4468.45	5442.55	1.22	3.5 × 10^−22^	1.4 × 10^−20^	up
40	miR-21-5p	7391.90	9253.61	1.25	1.3 × 10^−46^	8.1 × 10^−45^	up
41	miR-378a-3p	6381.27	8094.68	1.27	1.9 × 10^−45^	9.8 × 10^−44^	up
42	miR-145-5p	280.35	367.30	1.31	7.3 × 10^−4^	7.9 × 10^−3^	up
43	miR-150-5p	232.95	327.95	1.41	7.0 × 10^−5^	9.6 × 10^−4^	up
44	miR-486	1157.86	2573.86	2.22	0	0	up
45	miR-133a-3p	900.15	5278.65	5.86	0	0	up
46	miR-206-3p	4.26	25.52	5.99	1.9 × 10^−4^	2.4 × 10^−3^	up
47	miR-1b	27.67	168.76	6.10	9.3 × 10^−26^	4.3 × 10^−24^	up
48	miR-1-3p	14.47	96.03	6.63	7.2 × 10^−16^	2.0 × 10^−14^	up
49	miR-133b-3p	8.66	58.73	6.78	3.9 × 10^−10^	7.7 × 10^−9^	up
50	miR-133a-5p	2.98	24.93	8.367	2.7 × 10^−5^	4.1 × 10^−4^	up

**Table 3:** GO and KEGG enrichment analysis results of target genes of differently expressed miRNA

miRNAs	GO Enrichment				KEGG Pathway Enrichment					*p*- Value	FDR
	Different Genein All GO	Up Gene	Down Gene	All Gene in All GO	Different Genein All Pathway	Up Gene	Down Gene	All Gene in All Pathway	Score		
miR-1-3p	273	0	273	19,087	109	0	109	7597	>1	<0.05	<0.25
miR-206-3p	271	0	271	19,087	108	0	108	7597	>1	<0.05	<0.25
miR-133a-3p	222	0	222	19,087	80	0	80	7597	>1	<0.05	<0.25
miR-133b-3p	222	0	222	19,087	80	0	80	7597	>1	<0.05	<0.25
miR-133a-5p	129	0	129	19,087	52	0	52	7597	>1	<0.05	<0.25
Total different gene	558				240						

**Table 4:** Predicted target genes of differently expressed miRNAs.

Gene ID	Gene Symbol	NCBI Accession	Description	miRNAs
296566	Clic3	NM_001013080.2	chloride intracellular channel 3	rno-miR-1-3p
25321	Ghrhr	NM_012850.1	growth hormone releasing hormone receptor	rno-miR-1-3p
360622	Igfbp4	NM_001004274.2	insulin-like growth factor binding protein 4	rno-miR-1-3p
60326	Kcnv1	NM_021697.1	potassium channel, subfamily V, member 1	rno-miR-1-3p
291439	Mbd1	NM_001011924.1	methyl-CpG binding domain protein 1	rno-miR-1-3p
307271	Mex3c	NM_001107377.1	mex-3 homolog C (C. elegans)	rno-miR-1-3p
297525	Mkrn2	NM_001008314.1	makorin, ring finger protein, 2	rno-miR-1-3p
25482	Mras	NM_012981.2	muscle RAS oncogene homolog	rno-miR-1-3p
287962	Mrpl40	NM_001024865.1	mitochondrial ribosomal protein L40	rno-miR-1-3p
289482	Naa11(Ard1b)	NM_001024742.1	ARD1 homolog B (S. cerevisiae)	rno-miR-1-3p
296318	Ndrg3	NM_001013923.1	N-myc downstream regulated gene 3	rno-miR-1-3p
360967	Rnf185	NM_001024271.1	ring finger protein 185	rno-miR-1-3p
29503	Slc22a2	NM_031584.2	solute carrier family 22 (organic cation transporter), member 2	rno-miR-1-3p
114247	Slfn3	NM_053687.1	schlafen 3	rno-miR-1-3p
25033	Sstr1	NM_012719.2	somatostatin receptor 1	rno-miR-1-3p
362453	Crebl2	NM_001015027.1	cAMP responsive element binding protein-like 2	rno-miR-206-3p
289693	Dhx15	NM_001191597.1	DEAH (Asp-Glu-Ala-His) box polypeptide 15	rno-miR-206-3p
64124	Adgrl4(Eltd1)	NM_022294.1	EGF, latrophilin and seven transmembrane domain containing 1	rno-miR-206-3p
362862	Hsp90b1	NM_001012197.2	heat shock protein 90kDa beta (Grp94), member 1	rno-miR-206-3p
192361	Ppp1r2	NM_138823.2	protein phosphatase 1, regulatory (inhibitor) subunit 2	rno-miR-206-3p
54190	Rabep1	NM_019124.1	rabaptin, RAB GTPase binding effector protein 1	rno-miR-206-3p
192152	Tgoln2	NM_138840.2	trans-golgi network protein	rno-miR-206-3p
300857	Tmem30a	NM_001004248.1	transmembrane protein 30A	rno-miR-206-3p
113922	15-Sep	NM_133297.2	selenoprotein	rno-miR-133a-3p
300677	Atp5l	NM_212516.2	ATP synthase, H+ transporting, mitochondrial F0 complex, subunit G	rno-miR-133a-3p
24565	Abcc1	NM_022281.2	ATP-binding cassette, subfamily C (CFTR/MRP), member 1	rno-miR-133b-3p
25402	Casp3	NM_012922.2	caspase 3	rno-miR-133b-3p
298098	Pole3	NM_001007652.2	polymerase (DNA directed), epsilon 3 (p17 subunit)	rno-miR-133b-3p
295347	Rap1a	NM_001005765.1	RAP1A, member of RAS oncogene family	rno-miR-133b-3p
54296	Rgs7	NM_019343.1	regulator of G-protein signaling 7	rno-miR-133b-3p
29726	Slc22a5	NM_019269.1	solute carrier family 22 (organic cation/carnitine transporter), member 5	rno-miR-133b-3p
362691	Tmem178	NM_001004282.1	transmembrane protein 178	rno-miR-133b-3p
24179	Agt	NM_134432.2	angiotensinogen (serpin peptidase inhibitor, clade A, member 8)	rno-miR-133a-5p

**Table 5:** Target gene RT-PCR results of differently expressed miRNAs.

Arginine vs. Control	Target Genes	*n*	*p*-Value
No results	Slc22a2, Clic3, Kcnv1, Igfbp4, Sstr1, Ghrhr	6 vs. 6	
No difference	Mbd1, Naa11(Ard1b), Mras, Mrpl40, Rap1a, Rgs7, Abcc1, Pole3, Crebl2, Rabep1	6 vs. 6	
Up gene	Mkrn2, Slfn3, Mex3c, Rnf185, Ndrg3, Tgoln2, Dhx15, Slc22a5, Casp3, Tmem178a, E1td1(Adgrl4), Hsp90b1, Tmem30a, Ppp1r2, Agt	6 vs. 6	0.05
Down gene	Atp5l, 15-Sep	6 vs. 6	0.05

## Data Availability

Not applicable.

## Supplementary Materials

The following are available online at https://www.mdpi.com/2076-2615/11/2/535/s1, Table S1: Primer sequences of miRNA for qPCR. Table S2: Primer sequences of target genes for qPCR.
