# Supplementary material for: Arginine Alters miRNA Expression Involved in Development and Proliferation of Rat Mammary Tissue
Source: Animals (Basel). 2021 Feb 19;11(2):535. doi: 10.3390/ani11020535 (PMC7923093; doi:10.3390/ani11020535)
Supplement: Supplementary file 1 [file animals-11-00535-s001.pdf]

animals

Supplementary materials: Arginine Alters miRNA Expression Involved in Development and Proliferation of Rat Mammary Tissue

Gang Zhou [1,†], Qiaoyun Xu [2,†], Feifan Wu [2], Mengzhi Wang [2], Lianmin Chen [2,3,4], Liangyu Hu [2], Jingwen Zhao [2], Juan J. Loor [5] and Jun Zhang [2,*]

[1] Huaiyin Institute of Agricultural Sciences in Xuhuai Regio, Huaian 223000, Jiangsu Province, China; yzdxzg@163.com
[2] College of Animal Science and Technology, Yangzhou University, 88 South University Ave., Yangzhou 225009, China; jingkenxu@163.com (Q.X.); wffyzu@126.com (F.W.); mzwang@yzu.edu.cn (M.W.); lianminchen@yeah.net (L.C.); huyu1032@163.com (L.H.); zhaojingwen79@163.com (J.Z.)
[3] Department of Genetics, University of Groningen, University Medical Center Groningen, 9713 AV Groningen, The Netherlands
[4] Department of Pediatrics, University of Groningen, University Medical Center Groningen, 9713 AV Groningen, The Netherlands
[5] Department of Animal Sciences and Division of Nutritional Sciences, University of Illinois, 1207 W Gregory Drive, Urbana, IL 61801, USA; jloor@illinois.edu
* Correspondence: yzu7788@163.com; Tel.: +86-189-1213-9777
† These authors contributed equally.

Citation: Zhou, G.; Xu, Q.; Wu, F.; Wang, M.; Chen, L.; Hu, L.; Zhao, J.; Loor, J.J.; Zhang, J. Arginine Alters miRNA Expression Involved in Development and Proliferation of Rat Mammary Tissue. *Animals* **2021**, *11*, 535. https://doi.org/10.3390/ani11020535

Academic Editor: Johnny Roughan
Received: 11 January 2021
Accepted: 4 February 2021
Published: 19 February 2021

Publisher's Note: MDPI stays neutral with regard to jurisdictional claims in published maps and institutional affiliations.

Copyright: © 2021 by the authors. Submitted for possible open access publication under the terms and conditions of the Creative Commons Attribution (CC BY) license (http://creativecommons.org/licenses/by/4.0/).

Table S1. Primer sequences of miRNA for qPCR.

miRNA	Primer Sequences (5′-3′)
U6(F)	CGCTTCGGCAGCACATATAC
U6(R)	TTCACGAATTTGCGTGTCAT
rno-miR-133a-3p	TGGTCCCCTTCAACCAGC
rno-miR-486	TCCTGTACTGAGCTGCCCC
rnomiR-1b	GGCGTGGAATGTAAAGAAGTATGTAT
rnomiR-1-3p	GGGTGGAATGTAAAGAAGTGTGTAT
rnomiR-133b-3p	TTTGGTCCCCTTCAACCATA
rnomiR-133a-5p	GAGCTGGTAAAATGGAACCAAAT
rnomiR-206-3p	TGGAATGTAAGGAAGTGTGTGG

Table S2. Primer sequences of target genes for qPCR.

mRNAs	Primer Sequences (5′-3′)	Genebank ID
GAPDH	F:GGGTCATCATCTCTGCACCT<br>R:GGTCATAAGTCCCTCCACGA	XM_576394
Mbd1 (methyl-CpG binding domain protein 1)	F:GTAAGAGAGGCCCGAGGGTA<br>R:TCGCTGATGTGCAGTTCTGT	NM_001011924.1
Naa11 (N(alpha)-acetyltransferase 11)	F:GGCCCTGCACCTGTATTCTA<br>R:AGCTGCCTCCTCAGCTCAT	NM_001024742.1
Mkrn2 (makorin ring finger protein 2)	F:GGTTTGGCATTCTCTCCAAC<br>R:TATCACACGGCACTCTGGAC	NM_001008314.1
Mras (muscle RAS oncogene homolog)	F:CGTGCCTCTGACACAAACATA<br>R:CCGGACCAATAACGGTGA	NM_012981.2
Slc22a2 (solute carrier family 22, member 2)	F:GATGGAGTTCCCACTGGTTG<br>R:TGGCCTCTGCATATTCTCG	NM_031584.2
Clic3 (chloride intracellular channel 3)	F:ACGATGCCCTGTATCAGCAG<br>R:TTCAGAAAGGTGTGGCTCCT	NM_001013080.2
Slfn3 (schlafen 3)	F:TGTCCCAACAGTCCCTAAACA<br>R:CCAGTTAGAGGCCAGCGTTA	NM_053687.1
Kcnv1 (voltage-gated modifier subfamily V, member 1)	F:AGTGTTCCTTGTGCATGGTG<br>R:AATAGGCAAGGCCAAGACAA	NM_021697.1
Igfbp4 (insulin-like growth factor binding protein 4)	F:CCCTGTTTGTGGTGACTGG<br>R:GGGTTAGAGAGGTTGGGACA	NM_001004274.2
Mrpl40 (mitochondrial ribosomal protein L40)	F:GTTTGCAGAAGGCACCTGAT<br>R:GGGAAACTTTGCAGATGACC	NM_001024865.1
Rnf185 (ring finger protein 185)	F:ACATAAATGATGGGCGACCT<br>R:GGTCCTGTCTTAGGCGATCA	NM_001024271.1
Sstr1 (somatostatin receptor 1)	F:TGGAAGAACCTGGACGAAAC<br>R:ACAGTCCCACCAAGCATACC	NM_012719.2
Mex3c (mex-3 RNA binding family member C)	F:GCACGACTGTGTGATTTGCT<br>R:GGACATGATGGCGTTCTCTT	NM_001107377.1
Ghrhr (growth hormone releasing hormone receptor)	F:TCTGATCCCTTCCCACCATA<br>R:GAGGGCTACAATGGAGATGC	NM_012850.1
Ndrg3 (NDRG family member 3)	F:AATTCTCGCCTCGACCCTAT<br>R:CTGGCAGATGGTATGTAGCC	NM_001013923.1
Rap1a (RAP1A, member of RAS oncogene family)	F:AAGAACCTGAGGGCAGTGGT<br>R:TACGGCCAGCAAACTAAGGT	NM_001005765.1
Rgs7 (regulator of G-protein signaling 7)	F:CACTGAGGGCCAGCACTAAC<br>R:CTACAAGGTATGGGCGATGG	NM_019343.1
Slc22a5 (solute carrier family 22, member 5)	F:GTGGGACCTGGTGTGTAAGG<br>R:ACATTCTTGCGACCAAACCT	NM_019269.1
Casp3 (caspase 3)	F:TACAGAGCTGGACTGCGGTA<br>R:TAGTAACCGGGTGCGGTAGA	NM_012922.2
Abcc1 (ATP-binding cassette, subfamily C, member 1)	F:ACTTGTGAGGCTCCCTGAAA<br>R:GAGGCAGTGGCTTGTGTTT	NM_022281.2
Tmem178a (transmembrane protein 178A)	F:GTATGACTGCCTGCATCGAA<br>R:TTTGTGCTTGGAACCCTGAT	NM_001004282.1
Pole3 (polymerase, epsilon 3, accessory subunit)	F:GTGGCTTAAACTTGGCGGTA<br>R:CAGCAGCCATCATCTGAGAG	NM_001007652.2
Crebl2 (cAMP responsive element binding protein-like 2)	F:GCAATGGACCAAGGGAAA<br>R:CGAATTGGTGTTAGCGTCT	NM_001015027.1
Tgoln2 (trans-golgi network protein 2)	F:GCCTTATCTTATGGCGTTGG<br>R:AACTGGGTTCTTGGGAAGGA	NM_138840.2
Adgrl4 (adhesion G protein-coupled receptor L4)	F:TAGGGCCAGCGTGTCTAATC<br>R:TGGCGCAAGACCTTATGTT	NM_022294.1

Dhx15 (DEAH (Asp-Glu-Ala-His) box helicase 15)	F:AAACGTCTTCAGCTCCCTGT<br>R:TACCAGACCCAGTCTCACCA	NM_001191597.1
Hsp90b1 (heat shock protein 90, beta, member 1)	F:TCCGCCTCAGCTTAAACATT<br>R:TCCTGCTCGGAGTCATCTG	NM_001012197.2
Rabep1 (rabaptin, RAB GTPase binding effector protein 1)	F:AGACCGGGAGATAGCTGACC<br>R:CATCACGACAGAGCGAAGC	NM_019124.1
Tmem30a (transmembrane protein 30A)	F:TCCCGAGATGATAGCCAGTT<br>R:ACATGGAGCAATCGGTTTGT	NM_001004248.1
Ppp1r2 (protein phosphatase 1, regulatory (inhibitor) subunit 2)	F:TGGACGTGAGGGACTCTTCT<br>R:CCTGCCACAATACGGCTACT	NM_138823.2
Agt (angiotensinogen)	F:CTGAGCAGTCCGTTCCTGTT<br>R:AGGAGGCATCACACCACATT	NM_134432.2
Atp5l (ATP synthase, H+ transporting, mitochondrial Fo complex, subunit G)	F:CCCTTGGTGAAATCCCTACA<br>R:AACCATTCAGCACAGCTTCC	NM_212516.2
Sep15 (selenoprotein 15)	F:GGGCAGAGTTCTCGTCAGAG<br>R:TTTCAAACTGCGCTTCTTCC	NM_133297.2
